# Autophagy Inhibition Enhances Daunorubicin-Induced Apoptosis in K562 Cells

**DOI:** 10.1371/journal.pone.0028491

**Published:** 2011-12-02

**Authors:** Weidong Han, Jie Sun, Lifeng Feng, KaiFeng Wang, Da Li, Qin Pan, Yan Chen, Wei Jin, Xian Wang, Hongming Pan, Hongchuan Jin

**Affiliations:** 1 Department of Medical Oncology, Sir Run Run Shaw Hospital, College of Medicine, Zhejiang University, Hangzhou, Zhejiang, China; 2 Laboratory of Cancer Biology, Biomedical Research Center, Sir Run Run Shaw Hospital, College of Medicine, Zhejiang University, Hangzhou, Zhejiang, China; Ludwig-Maximilians University, Germany

## Abstract

Anthracycline daunorubicin (DNR) is one of the major antitumor agents widely used in the treatment of myeloid leukemia. Unfortunately, the clinical efficacy of DNR was limited because of its cytotoxity at high dosage. As a novel cytoprotective mechanism for tumor cell to survive under unfavorable conditions, autophagy has been proposed to play a role in drug resistance of tumor cells. Whether DNR can activate to impair the sensitivity of cancer cells remains unknown. Here, we first report that DNR can induce a high level of autophagy, which was associated with the activation of extracellular signal-regulated kinase 1/2 (ERK1/2). Moreover, cell death induced by DNR was greatly enhanced after autophagy inhibition by the pharmacological inhibitor chloroquine (CQ) and siRNAs targeting Atg5 and Atg7, the most important components for the formation of autophagosome. In conclusion, we found that DNR can induce cytoprotective autophagy by activation of ERK in myeloid leukemia cells. Autophagy inhibition thus represents a promising approach to improve the efficacy of DNR in the treatment of patients with myeloid leukemia.

## Introduction

Myeloid leukemia is a heterogeneous group of diseases characterized by uncontrolled proliferation of neoplastic hematopoietic precursor cells and impaired production of normal hematopoiesis leading to neutropenia, anemia, and thrombocytopenia [1]. Anthracycline daunorubicin (DNR) is one of the major antitumor agents widely used in the treatment of myeloid leukemia. The toxicities of DNR include cardiac toxicity, renal toxicity, severe myelosuppression, et al [2], [3], [4]. Cytotoxicities induced by DNR are mainly caused by drug-induced damage to DNA and production of free radicals which are initiated by reactive oxygen species (ROS) [5], [6]. Since the dosage used is critical to the incidence of DNR–induced toxicities, it would be important to reduce the dosage of DNR by increasing the sensitivity of leukemia cells to DNR.

Macroautophagy (hereafter referred to as autophagy) is a degradative process in eukaryotic cells that results in the breakdown of intracellular material within lysosomes [7], [8]. Under cellular stress conditions such as nutrient-deprivation, DNA damage and elevation of intracellular ROS, autophagy could be activated to provide an alternative source of energy to enable cell survival [9], [10], [11]. Excessive or unquenched autophagy, however, can lead to type II programmed cell death (PCD II), which is morphologically distinct from apoptosis and usually caspase independent [12]. In tumor cells, the role of autophagy may depend on the type of tumors, the stage of tumorigeneses, the nature and extent of the insults. Autophagy was upregulated during the later stages of cancer development so that cancer cells could survive in the central areas of the tumour which are usually poorly vascularized and lack of nutrient and oxygen [13], [14], [15]. A number of anticancer therapies, including DNA-damaging chemotherapeutic drugs, have been observed to induce the accumulation of autophagosomes in tumor cell lines and inhibition of autophagy by pharmacologic inhibitor or genetic knockdown of phylogenetically conserved autophagy-related genes, such as Atg5 and Atg7, usually enhanced drug-induced cytotoxicities [12]. In our previous research, we found that gefitinib induced autophagy in lung cancer cells and inhibiting autophagy increased the sensitivity of lung cancer cells to gefitinib, suggesting a novel approach to enhance target therapy of lung cancer [16].

In the present study, we demonstrated that DNR induced autophagy in myeloid leukemia cells by ERK1/2 activation. And DNR-induced apoptosis was enhanced after the inhibition of autophagy, suggesting a novel and promising strategy to increase the clinical efficacy of DNR for the treatment of myeloid leukemia.

## Materials and Methods

### Reagents and antibodies

Daunorubicin (DNR) and chloroquine (CQ) were purchased from J&K chemical Ltd., China. Monodansylcadaverine (MDC), U0126 and Hoechst were purchased from Sigma-Aldrich. Primary antibodies against microtubule-associated protein 1 light chain 3 (LC3), Atg5, Atg7, Beclin-1, caspase 3, caspase 9, PARP, GAPDH, non-phospho- or phospho-MEK1/2 and ERK1/2 were from Cell Signaling Technology, Inc.. The secondary antibodies were HRP conjugated anti-rabbit and anti-mouse IgG (Cell Signaling Technology, Inc.).

### Cell cultures and transfection

K562 were bought from cell bank (Chinese Academy of Sciences). Cells was maintained in RPMI 1640 supplemented with 10% (v/v) FBS. Stock solution of U0126 was prepared in dimethyl-sulphoxide (DMSO, Sigma) and diluted with medium before use. Final concentration of DMSO was <0.1%. The tfLC3 tandemly tagged with GFP and mRFP construct were obtained from Addgene. Transfection was performed via electroporation with a Nucleofection system (Amaxa, Inc.) according to the manufacturer's instructions. Stable transformants were selected in complete medium containing 500 µg/ml G418 (Sigma).

### Cytotoxicity assay

The cytotoxicity of chemicals against K562 cells was determined by MTT assay. Cells were seeded into 96-well plates and treated with chemicals with different concentrations. After 48-h incubation, 20 µl MTT (5 mg/ml) was added into each well for 4-h incubation and 150 µl DMSO was added into each well in order to solubilize the blue-purple crystals of formazan afterwards. The absorbance was then measured using a model ELX800 Micro Plate Reader (Bio-Tek Instruments, Inc.) at 570 nm.

### MDC-staining and Hoechst-staining

MDC was stored at−20°C under desiccant. A fresh stock solution of 5 mM MDC was made in 1∶1 DMSO immediately prior to use. At 1 hour before fixation, fresh medium that contained either 10 µM MDC in DMSO or an equivalent volume of DMSO was added to the cells. The cells were fixed using a freshly made 4% formaldehyde solution in PBS for 10 min. at room temperature and imaged immediately.

For identification of cells with nuclear changes typical of apoptosis, cells were stained with 5 µg/ml of Hoechst 33342 for 10 min. at 4°C in the dark and analyzed using a UV microscope.

### Immunofluorescence and confocal microscopy

Cells were collected, fixed and permeabilized with 1% CHAPS buffer (150 mM NaCl, 10 mM HEPES, 1.0% CHAPS) at room temperature for 10 min, incubated with anti-LC3 for 2 h at room temperature, and washed with PBS, incubated for another 45 min with FITC-conjugated goat anti-rabbit IgG (Beyotime, A0562). Then, cell nuclei were stained by DAPI (Sigma, D9564). Samples were examined under a Zeiss LSM 710 confocal microscope system (Carl Zeiss, Germany). Image was processed with ZEN LE software.

For evaluating tandem fluorescent LC3 puncta, stable transformants after treatment were washed with PBS, fixed with 4% paraformaldehyde, mounted with DAPI and viewed under the confocal microscope.

### Electron microscopy

Treated cells were washed and fixed for 30 min in 2.5% glutaraldehyde. The samples were treated with 1.5% osmium tetroxide, dehydrated with acetone and embedded in Durcupan resin. Thin sections were poststained with lead citrate and examined in the TECNAI 10 electron microscope (Philips, Holland) at 60 kV.

### Western blot analysis

Western blotting was carried out as previously reported [17]. Briefly, proteins were resolved by SDS-polyacrylamide gel electrophoresis, transferred to a PVDF membrane and then detected by the proper primary and secondary antibodies before visualization with a chemiluminescence kit (Pierce). The visualization was done with Image Quant LAS-4000 (Fujifilm, Tokyo, Japan).

### RNA interference

K562 cells were transfected with either nonspecific or Atg5/Atg7 RNA interference (All siRNAs were from Qiagen, RNAi; final concentration, 100 nmol/L) via LipofectAMINE 2000 (invitrogen) according to the manufacturer's instructions. Cells were then incubated for 48 h prior to Western blot or MTT assay.

For Beclin-1 RNA interference, two siRNA oligonucleotides targeting Beclin-1 were bought from Cell Signaling Tech (#6222 & 6246). A nonspecific oligo that is not complementary to any human genes was used as a negative control. To transfect K562 cells, HiPerFect reagent (QIAGEN) was used following the manufacturer's protocol.

### Statistical analyses

Unless otherwise stated, data were expressed as the mean ± SD and analyzed by Student's t test.

## Results

### DNR induced apoptosis in K562 cells

**Figure 1:**
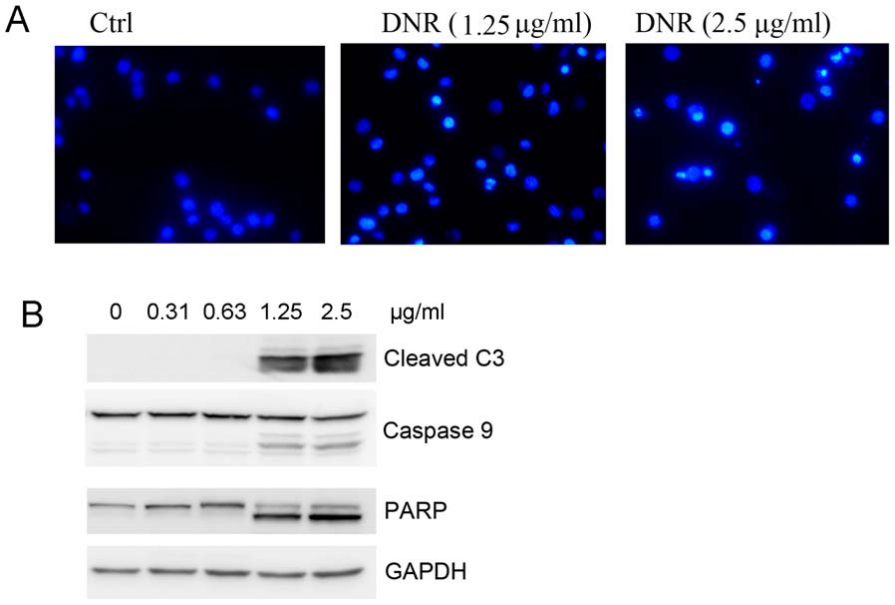
DNR induced apoptosis in K562 cells. A. K562 cells were treated with DNR for 24 hours, and stained by Hoechst. B. Cells were treated with varying concentration of DNR for 24 hours and examined for activation of caspases 3, 9 and cleavage of PARP with Western Blotting analysis.

K562 cells were incubated in the presence of varying concentration of DNR for 24 hours and subjected to morphological and biochemical examinations. A dose-dependent nuclear morphology changes were observed (Fig. 1A). Cells had typical morphology of apoptosis such as nuclear chromatin condensation and nuclear fragmentation. Biochemically, caspase 3 and 9 were activated by DNR in a dose-dependent manner (Fig. 1B). Consistently, the level of cleaved product of caspases substrate Poly ADP-ribose Polymerase (PARP) was correlated with the activation of caspases (Fig. 1B). Collectively, these results demonstrated that DNR could induce apoptosis in K562 cells.

### DNR activated autophagy in K562 cells

**Figure 2:**
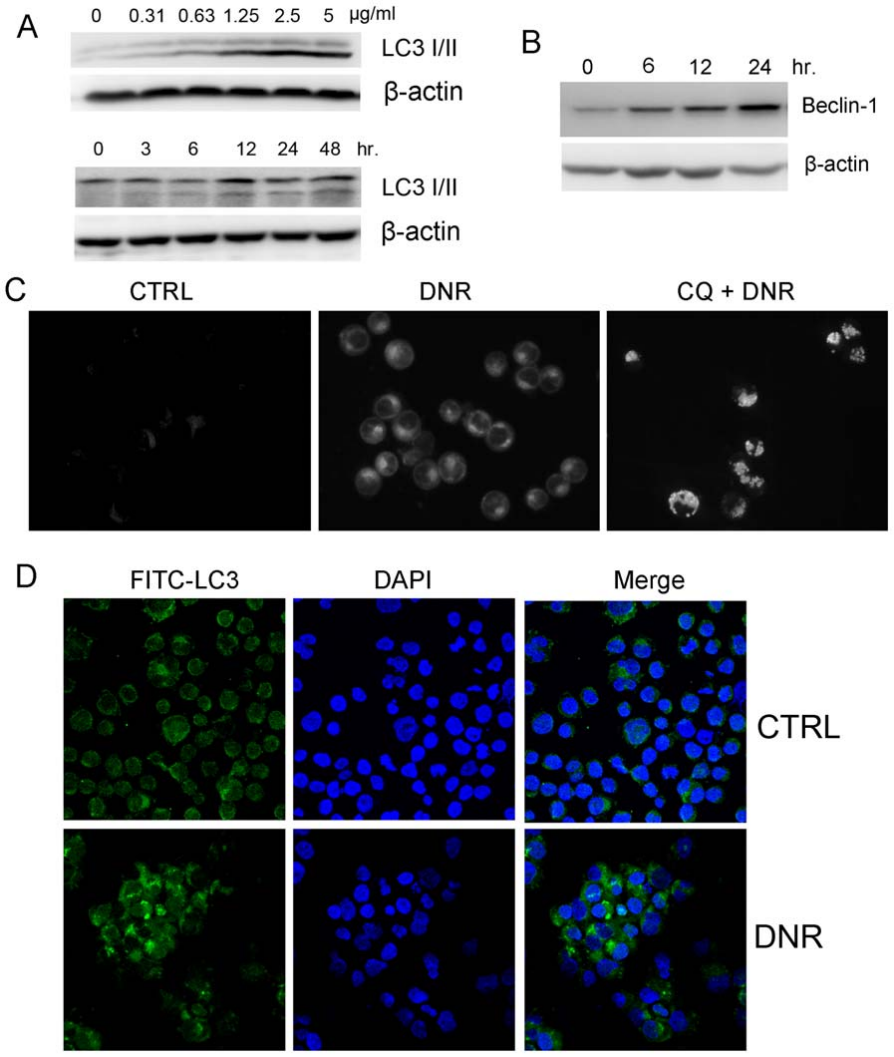
DNR induced autophagy in K562 cells. A. K562 cells were incubated with varying concentrations of DNR for 24 hours or incubated with 1.25 µg/ml DNR for appropriate intervals. The switch of LC3-I to LC3-II was detected by immunoblotting. B. Immunoblotting for Beclin-1 using lysates from K562 treated with 1.25 µg/ml DNR for appropriate intervals. C. MDC staining of K562 treated with DNR. Cells treated with 1.25 µg/ml DNR in the presence or absence of 5 µM CQ for 24 hours were stained with MDC. D. K562 cells were treated with 1.25 µg/ml DNR for 24 hours, and stained by indrect immunofluorescence as described in Marterial & Methods. The distribution of endogenous LC3 was monitored at confocal microscope.

To evaluate the activation of autophagy by DNR, the conversion of LC3-I into LC3-II before and after DNR treatment was first determined by western blotting analysis. DNR can induce the switch of LC3-I to LC3-II in the dose and time dependent manner in K562 cells, indicating that autophagy might be activated by DNR (Fig. 2A). Consistently, Beclin-1, a regulator of autophagic pathway, was significantly up-regulated in DNR-treated cells (Fig. 2B). Moreover, DNR treated cells were stained with monodansylcadaverine (MDC), a fluorescent dye selectively recognizing autophagosomes and autolysosomes [18]. As shown in Figure 2C, DNR treated cells revealed a diffuse punctuate pattern which indicates the activation of autophagy while the control cells show faint fluorescent. After the treatment of Chloroquine (CQ) which can disrupt the function of lysosomes and inhibit autophagy at late stage, DNR treatment resulted in the accumulation of large MDC-positive vesicles, confirming the activation of autophagy by DNR (Fig. 2C). To further confirm it, the distribution of endogenous LC3-II in cells before and after DNR treatment was monitored by indirect immunofluorescence staining. Indeed, specific punctate distribution of endogenous LC3-II was seen in the K562 cells treated with DNR (Fig, 2D).

**Figure 3:**
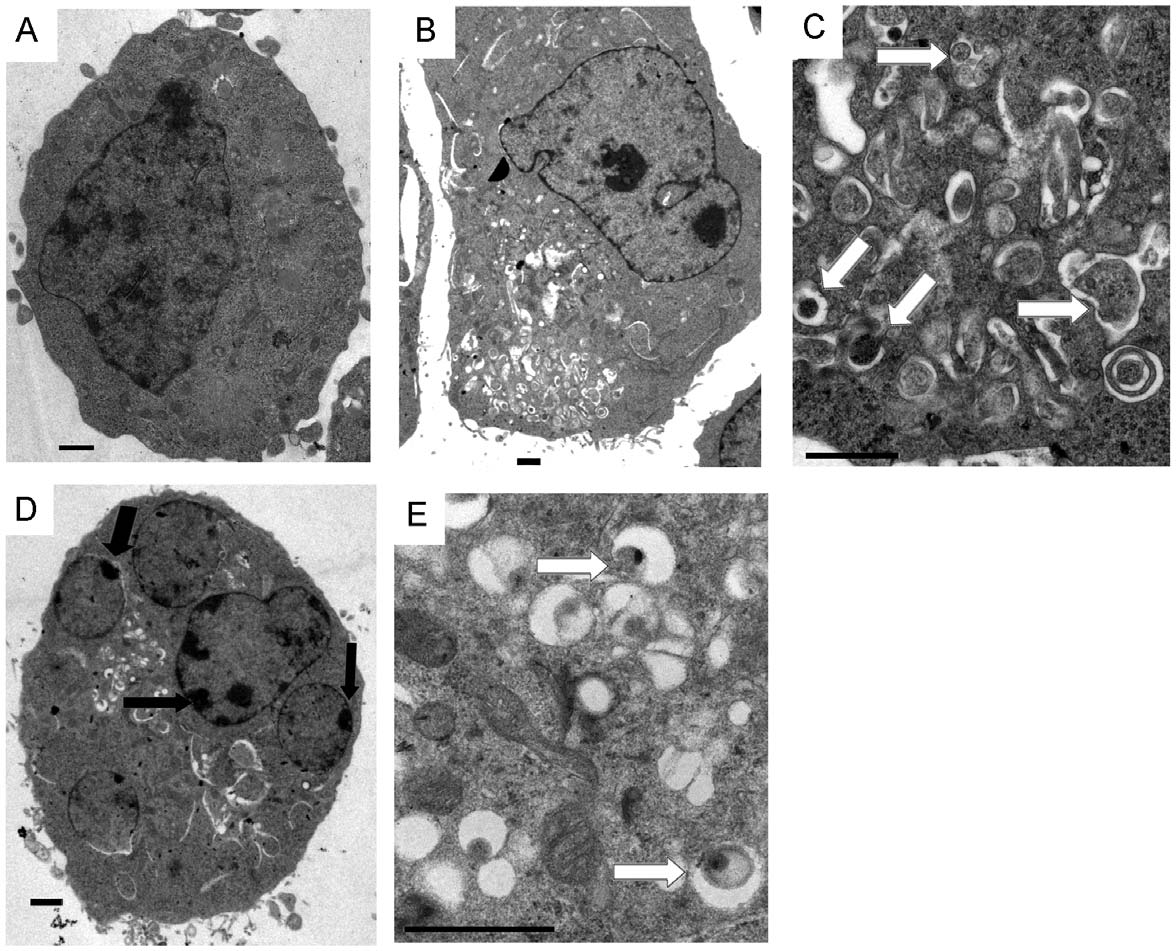
TEM depicted ultrastructures of K562 cells treated with DNR. Cells treated without (A) or with 2.5 µg/ml DNR for 6 hours (B, low power; C, high power) and 12 hours (D, low power; E, high power) were subjected to TEM analysis. Autophagosomes were highlighted by white arrows in C and E while apoptotic changes such as chromatin condensation and nuclear fragmentation were highlighted by black arrows in D. Bar  = 1 µm.

Furthermore, ultrastructural analysis by electron microscopy further confirmed that large autophagic vacuoles with typical double-layer membrane containing organelle remnants presented in DNR-treated K562 cells rather than untreated cells (Fig. 3). Besides, K562 cells treated with 2.5 µg/ml DNR for 12 hours displayed a typical nuclear morphology of apoptosis such as chromatin condensation and nuclear fragmentation (Fig. 3D).

### Blockage of autophagy enhanced DNR-induced K562 cell death

**Figure 4:**
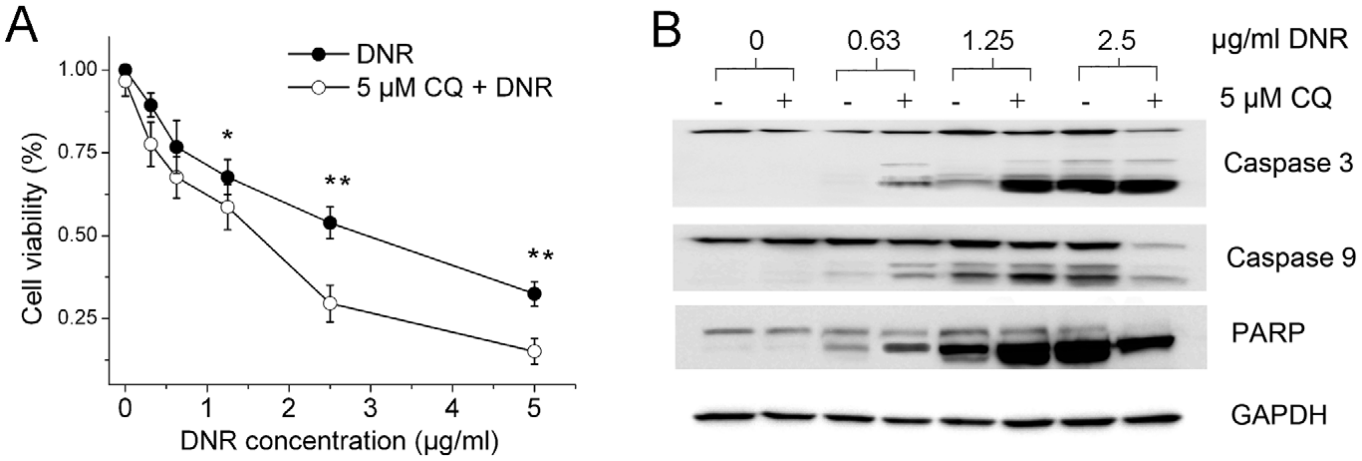
Autophagy inhibitor CQ enhanced DNR-induced apoptosis in K562 cells. A. Cells were treated with varying concentration of DNR for 48 hours in the presence or absence of 5 µM CQ. Cell viability was then measured by MTT assay. B. Immunoblotting of caspase 3, caspase 9 and PARP using lysates from K562 cells as treated in A. Data were expressed as the mean ± SD, and analyzed by Student's t test. *, P<0.05 and **, P<0.01.

Many studies have demonstrated that autophagy may serve as a protective response preventing tumor cells from therapy-induced cell death [10]. To test this consumption, we compared the growth inhibitory effect of DNR on cancer cells before and after pharmacological and genetic inhibition of autophagy. As shown in Figure 4A, CQ failed to inhibit K562 cell growth by its own. However, it significantly inhibited the autophagy induced by DNR (Figure S1) and augmented growth inhibition induced by DNR as well as DNR-induced caspase activation (Fig. 4B)[19].

**Figure 5:**
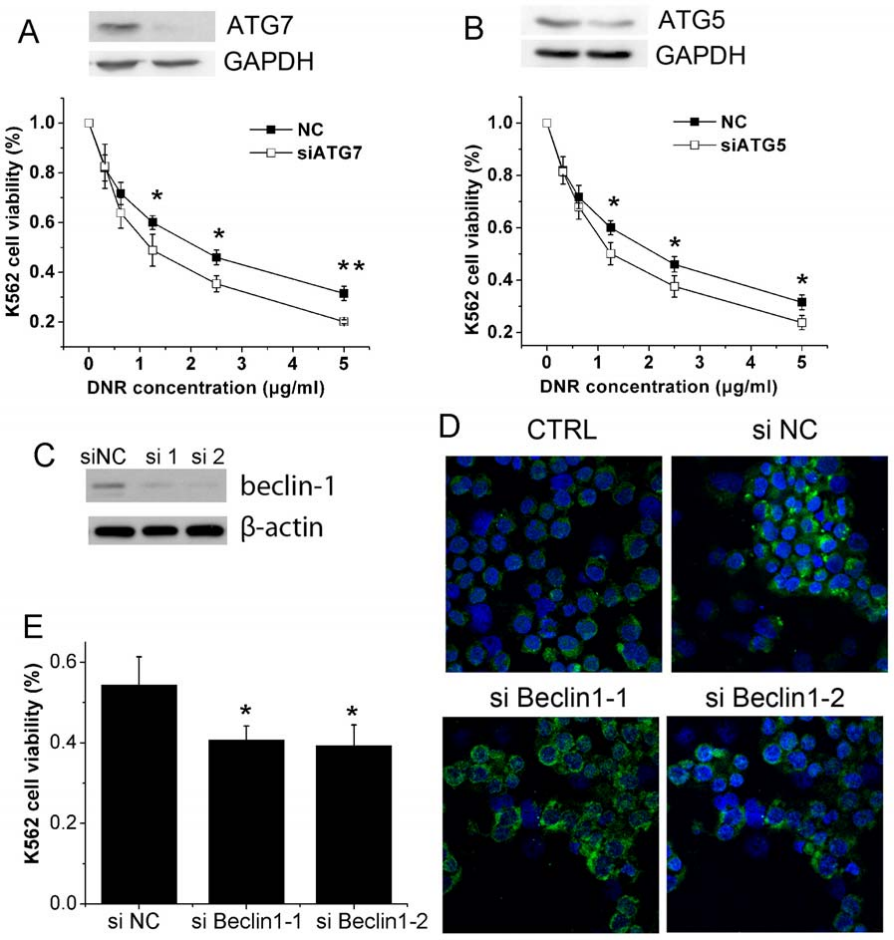
Depletion of autophagy genes enhanced DNR-induced apoptosis in K562 cells. A & B, Cells transiently transfected with negative control siRNA, Atg7 or Atg5 siRNA were treated with varying concentration of DNR for 48 hours. Cells viability was measured by MTT assay and the expression of Atg5 and Atg7 were determined by Immunoblotting. NC, negative control; C, Cells were transiently transfected with negative control siRNA or beclin-1 targeting siRNAs, and the expression of beclin-1 was determined by western blot. D, Cells were treated with 1.25 µg/ml DNR for 24 hours, and stained by indrect immunofluorescence as described in Marterial & Methods. The distribution of endogenous LC3 was monitored at confocal microscope. Green, FITC labelled LC3; blue, DAPI stained nuclei. E, Cells transiently transfected with beclin-1 targeting siRNAs were treated with 1.25 µg/ml DNR for 48 hours, and the viability was measured by MTT assay. Data were expressed as the mean ± SD, and analyzed by Student's t test. * P<0.05, ** P<0.01.

Autophagy is tightly regulated by several highly conserved molecules called ATGs [11]. Since ATGs are essential to the activation of autophagy, siRNAs specific to human Atg5 and Atg7 were used to block autophagy after DNR treatment (Fig. 5A and B). Consistent with the pharmacological inhibition of autopahgy, growth inhibition induced by DNR was markedly enhanced after autophagy was blocked by the knockdown of Atg5 and Atg7 (Fig. 5A and B), confirming that DNR-activated autophagy is cytoprotective.

As mentioned above, Beclin-1, a regulator of autophagic pathway, was significantly up-regulated in DNR-treated cells (Fig. 2B). This result prompted us to investigate if knockdown of Beclin-1 will impair DNR induced autophagy? As expected, knockdown of beclin-1 could significantly decrease the number of puncta LC3 induced by DNR (Fig. 5C&D). And we also measured the cytotoxiciy of DNR against K562 cells transfected with beclin-1 siRNAs. Inhibition of autophagy by knockdown of beclin-1 sensitized K562 cells to DNR (Fig5. E).

### DNR induced autophagy by activating of MEK/ERK signaling pathway

**Figure 6:**
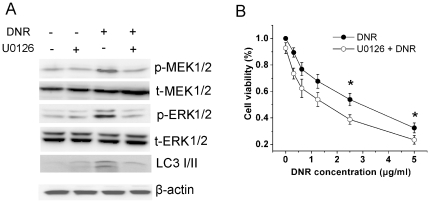
Activation of autophagy by DNR was MEK/ERK-dependent. A, K562 cells were treated with 1.25 µg/ml DNR for 24 hours in the presence or absence of 5 µM U0126 and the switch of LC3-I to LC3-II as well as the phosphorylation of MEK 1/2 and ERK1/2 were determined by Immunoblotting. B, Cells were treated with varying concentration of DNR for 48 hours in the presence or absence of 5 µM U0126. Cells viability was measured by MTT assay. Data were expressed as the mean ± SD, and analyzed by Student's t test. * P<0.05.

It have been reported that autophagy was associated with MAPK signaling pathway [20], [21], [22], [23]. Therefore, we determined the relevance of this pathway to DNR-induced autophagy. Indeed, phosphorylation of MEK1/2 and ERK1/2 were significantly increased after DNR treatment (Fig. 6A). Pre-treatment of K562 cells with the MEK1/2 inhibitor U0126 resulted in the decrease of LC3-II levels (Fig. 6A), indicating the dependence of autophagy on the activation of MAPK signaling. In consistence with previously finding (Fig 4 and 5), U0126 significantly augmented DNR-induced growth inhibition of K562 cells (Fig. 6B).

## Discussion

Anthracycline daunorubicin is one of the major antitumor agents widely used in the treatment of myeloid leukemias. Cytotoxicity mediated by DNR is generally thought to be the result of drug-induced damage to DNA. Interestingly, previous studies demonstrated that DNR triggered both apoptotic and survival pathways. The phospholipase C–dependent DAG/raf-1/MEK cascade and DAG independent PI3K/PKCζ cascade play a significant role in DNR induced cellular survival pathways [24]. As well known, both raf-1/MEK and PI3K cascade are involved in the autophagy signaling [11], [25]. In addition to DNR, many other DNA-damaging drugs have been confirmed to activate autophagy in various tumor cell lines and inhibition of autophagy by pharmacologic inhibitors or genetic knockdown of critical autophagy-related genes such as Atg5 and Atg7 could enhance the anti-cancer effect of chemotherapeutic drugs [10]. However, very little was known about the relevance of autophagy in the response of myeloid leukemia cells to DNR treatment. This study, for the first time, demonstrated that DNR can induce cytoprotective autophagy in K562 cells by the activation of MEK/ERK signaling pathway.

Autophagy is morphologically characterized by the appearance of “double-membrane” vacuoles, autophagosomes, in the cytoplasm. In addition, LC3, the mammalian homologue of the yeast protein Apg8p, was found to be a specific biochemical marker for autophagy. Newly synthesized LC3 termed LC3-I is evenly distributed throughout the cytoplasm. Upon the induction of autophagy, some LC3-I is converted into LC3-II which is tightly bound to the autophagosomal membranes, forming ring-shaped structures in the cytosol. We confirmed biochemically and morphologically that autophagy was activated by DNR in K562 cell lines (Fig. 2 and 3).

In addition, we found that DNR could induce both apoptosis and autophagy in K562 cells (Fig.3 D and E). Inhibition of autophagy enhanced the activation of caspases as well as the tumor inhibition induced by DNR (Fig. 4 and Fig. 5). Based on the EM results, we postulated that autophagy activated by DNR in K562 cells was probably earlier than apoptosis. When K562 cells treated with DNR for 6 hours, we could find numerous autophagical vacuoles with typical double-layer membrane containing organelle remnants in most of cells while the apoptotic changes of nuclei were less observed. However, chromatin condensation and nuclear fragmentation were easier to find only after K562 cells were treated with DNR for 12 hours. This result was consistent with previous reports that autophagy preceded apoptosis in many cancer cells treated with various cytotoxic drugs [26], [27], [28].

As one critical signaling pathways involved in tumorigenesis, MAPK signaling pathway are often activated in numerous types of cancer cells. Accumulating evidences indicated that the activation of ERK1/2 was associated with autophagy [22], [23], [29], [30], [31]. In our study, we found that DNR indeed activated MEK1/2 and ERK1/2. Inhibition of MEK1/2 by U0126 inhibited the shift from LC3-I to LC3-II, thus enhancing the cytotoxic effect of DNR. Previous studies indicted that the constant activation of MEK/ERK signaling in tumor cells could up-regulate Beclin-1, eventually activating autophagy [23]. Indeed, Belin 1 expression in K562 cells was increased after DNR treatment (Fig. 2B). Knockdown of beclin-1 could significantly decrease the number of puncta LC3 induced by DNR (Fig. 5C&D), and sensitized K562 cells to DNR treatment (Fig. 5E).

In summary, our results demonstrated that DNR could induce growth inhibition as well as cytoprotective autophagy in myeloid leukemia cells. Blocking autophagy by autophagy inhibitors or inhibitors of MAPK signaling is a promising therapeutic strategy to enhance tumor inhibition induced by DNR and probably other chemotherapeutic drugs.

## Supporting Information

Figure S1 **CQ inhibited DNR-induced autolysosomes formation.** K562 cells with stable expression of tfLC3 were treated with 1.25 µg/ml DNR for 24 hours in the presence or absence of 5 µM CQ. mRFP and GFP were monitored at confocal microscope as described in M&M. The red puncta that overlay with the green puncta and appear yellow in merged images are indicators of autophagosomes, whereas the free red puncta that do not overlay with the green puncta and appear red in merged images are indicative of autolysosomes [19].
(TIF)
