# Supplementary figures and images for: Autophagy Inhibition Enhances Daunorubicin-Induced Apoptosis in K562 Cells
Source: PLoS One. 2011 Dec 2;6(12):e28491. doi: 10.1371/journal.pone.0028491 (PMC3229606; doi:10.1371/journal.pone.0028491)

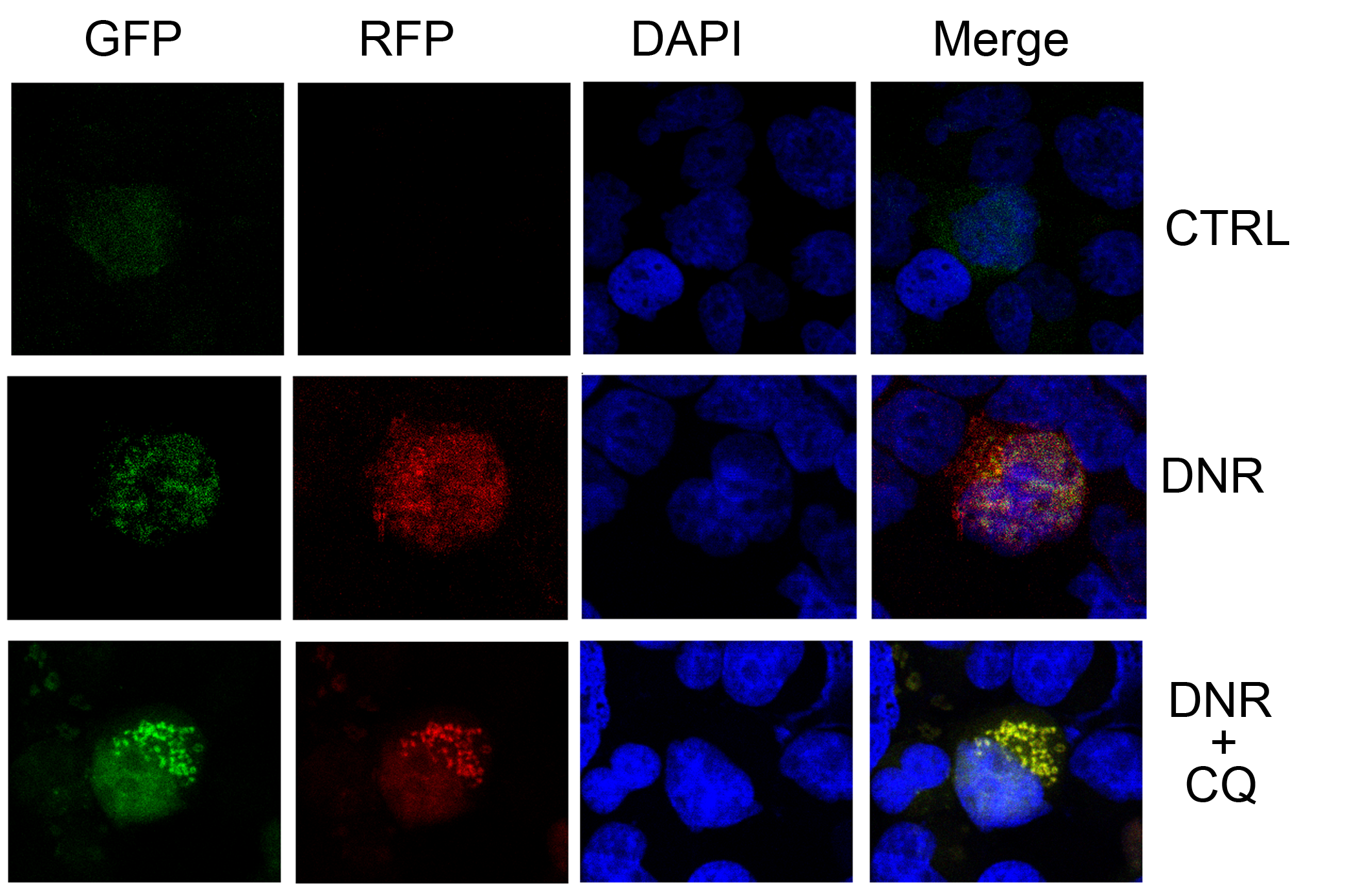

Supplement: Figure S1 — CQ inhibited DNR-induced autolysosomes formation. K562 cells with stable expression of tfLC3 were treated with 1.25 µg/ml DNR for 24 hours in the presence or absence of 5 µM CQ. mRFP and GFP were monitored at confocal microscope as described in M&M. The red puncta that overlay with the green puncta and appear yellow in merged images are indicators of autophagosomes, whereas the free red puncta that do not overlay with the green puncta and appear red in merged images are indicative of autolysosomes [19]. (TIF) [file pone.0028491.s001.tif]
